# Identification of a diagnostic metabolomic fingerprint in plasma for eosinophilic granulomatosis with polyangiitis

**DOI:** 10.1371/journal.pone.0343182

**Published:** 2026-05-12

**Authors:** Songsen Su, Yanfang Lin

**Affiliations:** 1 Department of Rheumatology, Quanzhou First Hospital Affiliated to Fujian Medical University, Quanzhou, Fujian, China; 2 Department of Pharmacy, Quanzhou First Hospital Affiliated to Fujian Medical University, Quanzhou, Fujian, China; University of Westminster - Regent Street Campus: University of Westminster, UNITED KINGDOM OF GREAT BRITAIN AND NORTHERN IRELAND

## Abstract

**Objective:**

Eosinophilic granulomatosis with polyangiitis (EGPA) was a rare systemic vasculitis characterized by eosinophilia, asthma, and necrotizing vasculitis. Metabolic dysregulation had been shown to participate in the pathogenesis of autoimmune diseases, but the plasma metabolic profile of EGPA remained unclear. This work was designed to systematically characterize the plasma metabolomic profiles of EGPA patients, identify differential metabolites that distinguish EGPA from bronchial asthma (BA), and explore their potential as biomarkers for differential diagnosis.

**Methods:**

Ten patients with EGPA, ten patients with BA, and ten age- and gender-matched healthy controls (HCs) were enrolled. Untargeted metabolomics based on liquid chromatography/mass spectrometry (LC/MS) was performed to analyze the metabolic profiles of the three groups. Differential metabolites were identified using VIP > 1 and P < 0.05. Kyoto Encyclopedia of Genes and Genomes (KEGG) pathway enrichment analysis was performed on the differential metabolites, with statistical significance defined as P < 0.05.

**Results:**

A total of 971 metabolites were differentially expressed between EGPA patients and HCs. KEGG pathway enrichment analysis identified 59 pathways, five of which were statistically significant (P < 0.05): caffeine metabolism; valine, leucine and isoleucine biosynthesis; alanine, aspartate and glutamate metabolism; arginine and proline metabolism; and glyoxylate and dicarboxylate metabolism. Comparison of EGPA with BA patients revealed 161 altered metabolites and 102 enriched pathways, three of which were significant (P < 0.05): caffeine metabolism, basal cell carcinoma, and Fc gamma R-mediated phagocytosis. Receiver operating characteristic (ROC) curve analysis demonstrated that 21 of the 24 metabolites identified from the five key EGPA-HC pathways exhibited strong diagnostic performance (area under the curve [AUC] > 0.8). Four metabolites (cholesterol, 5-acetylamino-6-formylamino-3-methyluracil, 5-acetylamino-6-amino-3-methyluracil, and 3-methylxanthine) showed high diagnostic potential (AUC > 0.8) for distinguishing EGPA from BA.

**Conclusion:**

This study revealed, for the first time, a distinct plasma metabolic profile in EGPA patients, with key pathways and candidate biomarkers identified. The metabolites with high diagnostic efficacy (AUC > 0.8) might serve as candidate diagnostic biomarkers for EGPA and its differentiation from BA. These observations provided novel insights into the metabolic basis of EGPA pathogenesis and might provide valuable references for the clinical management of this rare disease.

## Introduction

Eosinophilic granulomatosis with polyangiitis(EGPA), also known as Churg-Strauss syndrome(CSS), was a systemic inflammatory disease characterized by necrotizing vasculitis that predominantly affected small and medium-sized vessels [1–3]. The disease typically manifested as a triad of allergic asthma, significant peripheral eosinophilia, and multiple organ damage. The exact pathogenesis of EGPA remained to be clarified, but it was widely acknowledged that the activation of eosinophils, T cells, and neutrophils in EGPA, as well as the release of cytokines and inflammatory mediators, contributed to endothelial injury and granuloma formation. Studies had suggested that allergic rhinitis, vaccination, repeated injections of desensitizing agents, some drugs, and other antigenic stimuli might be the inducing factors of EGPA [4].

The clinical manifestations of EGPA were highly diverse and could affect multiple organ systems, including the peripheral nerves, heart, skin, kidneys, and gastrointestinal tract. The lungs were the most frequently and severely involved organ [5,6]. Due to the absence of specific clinical symptoms or laboratory findings, EGPA was often prone to misdiagnosis or diagnostic delay.In particular, EGPA typically developed in individuals with pre-existing asthma, which led to frequent misdiagnosis as asthma. Even for experienced rheumatologists, diagnosing EGPA posed a significant challenge due to its diverse clinical presentation, potential overlap with similar conditions such as asthma, and its association with sinusitis and eosinophilia.

Metabolomics had been gradually applied to personalized medicine and had achieved successful application in disease diagnosis and clinical treatment. This technology was applied to many fields including cancers, diabetes, coronary heart disease, and ophthalmic diseases [7–10]. Chen et al. employed metabolomics to examine the metabolic profiles of Adult-Onset Still’s Disease (AOSD) and identified 13 distinct metabolites involved in amino acid, fatty acid, phospholipid, taurine, and pentose phosphate metabolism [11]. Similarly, recent research had revealed notable distinctions in serum metabolites between patients with Behcet’s Disease (BD) and HCs, with linoleic acid (LA) and arachidonic acid (AA) identified as crucial biomarkers for BD diagnosis [12].

Metabolomic profiling could represent a powerful technique for the diagnosis of EGPA, however, metabolomic studies of EGPA had not been reported. Our study was designed to address these unresolved issues by studying the plasma metabolomic profile of patients with EGPA using an LC/MS-based metabolomics platform. We employed Principal Component Analysis (PCA) and Orthogonal Projections to Latent Structures Discriminant Analysis (OPLS-DA) to compare plasma metabolomic profiles between EGPA patients and HCs, as well as between EGPA patients and BA patients. Through this analysis, we sought to identify characteristic markers from many components to provide a basis for early diagnosis of EGPA.

## Materials and methods

### Study populations

Ten patients were recruited according to the inclusion criteria—the American College of Rheumatology (ACR) for EGPA, while ten patients with BA meeting the diagnostic standard of BA [13]. served as disease controls. All of the patients visited Quanzhou First Hospital Affiliated to Fujian Medical University, Fujian, China, with a recruitment period from 24/01/2024–25/10/2025. Healthy subjects matched for age, gender, and ethnicity served as controls and were recruited over the same period. Ethical approval for this study was granted by the Ethics Committee of Quanzhou First Hospital Affiliated to Fujian Medical University (Approval No. [2024] K142), and written informed consent was obtained from all patients before enrollment in the study.

### Sample preparation

Plasma samples collected from patients and healthy volunteers were transported on dry ice and stored at −80℃. Prior to the analysis, frozen plasma was defrosted and kept at 4℃. To precipitate proteins, the plasma (100 μL) was mixed with 400 μL of acetonitrile/methanol (1: 1 v/v) in a 1.5-mL tube. After being vortexed for 5 min and left to stand for 1 hour at −20℃, the mixture was centrifuged at 4000 × g for 15 min. The supernatant was transferred to a new Eppendorf tube and then evaporated to dry in a Speed Vac concentrator. Finally, the residues were resuspended in 100 μL mobile phase for LC/MS analysis.

### LC/MS analysis

The metabolomic analysis workflow, including sample preparation, LC/MS detection, metabolite identification, and statistical analysis, was performed with reference to previous studies [14,15]. UPLC Q-TOF/MS analysis was performed using the Nexera X2 system (Shimadzu Co., Japan) together with a Triple TOF 5600 quadrupole-time-of-flight mass spectrometer (AB SCIEX, USA). In brief, metabolites were separated using a ZORBAX Eclipse Plus C18 column (2.1 × 100 mm, 3.5 µm, Agilent, USA) maintained at 35℃. The volume of the sample injection was 3 μL for each run conducted in full loop injection mode, and the flow rate of the mobile phase was set as 0.5 mL/min. In RPLC mode, gradient elution was performed with the solvent system as follows: (A) 0.1% formic acid and water with (B) 0.1% formic acid in acetonitrile. The gradient program for mobile phase A was as follows: 0 min, 98% A; 13 min, 10% A; 16 min, 10% A; 16.1 min, 98% A; and 20 min, 98% A. Mass spectrometric analysis was performed with a Triple TOF 5600 + instrument with an ESI source. The data in positive and negative ionization modes were acquired for LC/MS analysis. The MS parameters are shown in Table 1.

**Table 1:** The MS parameters.

parameter	value
capillary voltage	3000-4500 V
cone gas	50 L/h
desolvation gas	600 L/h
source temperature	120 °C
desolvation temperature	500°C
scan range	50-1500 m/z

### Metabolites identification

The UPLC Q-TOF/MS data were preprocessed by MarkerView software, including retention time, peak discrimination, filtering, alignment, matching, and identification. Retention time (tR), m/z value and peak intensity were obtained. Feature annotations for the reversed-phase liquid chromatography streams were performed by matching MS1 and MS2 spectral against the Human Metabolome Database (HMDB; http://www.hmdb.ca/) Metabocard, PubChem descriptions, and KEGG pathways.

### Quality control and data processing

For metabolomics, we set quality control (QC) for normalization, and pooled QC samples were prepared by mixing equal amounts of plasma from all samples. The pretreatment of the QC samples was performed in parallel and was the same as the other samples. The QC samples were evenly inserted between each set of runs to monitor the stability of the analysis.

Compound identification was based on precise mass-to-charge ratio (M/z), secondary fragments, and isotopic distribution using HMDB, LipidMaps (V2.3) and Metlin, databases to do qualitative analysis. The extracted data were then further processed by removing any peaks with a missing value (ion intensity = 0) in more than 50% in groups, by replacing zero value by half of the minimum value, and by screening according to the qualitative results of the compound. A data matrix was combined from the positive and negative ion data.

Differential metabolites were selected with Fold Change (FC) values greater than 2.0 or lower than 0.5 and nominal P-values less than 0.05.

### Statistical analysis

Baseline characteristics were compared using the Student’s t-test for continuous variables and the Chi-square test for categorical variables. For non-normally distributed data, comparisons between groups were performed using the Mann-Whitney U test. Multivariate analysis was conducted using the MetaboAnalyst 4.0 program. PCA and OPLS-DA models were constructed to identify differential metabolites between the two groups. The metabolite ions were determined according to m/z, and HMDB was used for screening the potential biomarkers [16]. The molecular weight tolerance was set at ±0.05 Da. The area under the ROC curve was calculated for each potential biomarker. Differences were considered significant at P < 0.05. Enrichment analysis and pathway analysis were performed. SPSS version 22 was used for all other analyses.

## Results

### Characteristics of participants

The main characteristics of the participants were shown in Table 2. There were no significant differences in sex, age, BMI, albumin, triglyceride, LDL-C, HDL-C, glucose, AST and ALT levels among the three groups (all P > 0.05). Compared with HCs, patients with EGPA exhibited significantly higher levels of urea, uric acid, ESR, and CRP, as well as lower cholesterol levels (all P < 0.05). Compared to the BA group, the EGPA group had significantly higher levels of urea and uric acid, and significantly lower cholesterol. In contrast, the elevations in ESR and CRP in the EGPA group compared to the BA group were not statistically significant.

**Table 2:** Baseline characteristics of EGPA patients, BA patients, and HCs.

Characteristics	EGPA patients	BA patients	HCs
Number of samples	10	10	10
Age (mean, range)	62(42-71)	51(27-61)	56.7(44-67)
Gender (F/M)	(4/6)	(5/5)	(4/6)
BMI (kg/m²)	21.87 ± 1.87	21.46 ± 2.26	22.42 ± 2.78
Albumin (g/L)	40.99 ± 2.72	40.09 ± 4.85	39.33 ± 3.34
Triglyceride (mmol/L)	1.10 ± 0.31	1.23 ± 0.59	1.25 ± 0.61
Cholesterol (mmol/L)	4.38 ± 0.93^ab^	5.37 ± 0.95	5.40 ± 0.94
LDL-c (mmol/L)	2.63 ± 0.79	2.84 ± 0.85	2.82 ± 0.79
HDL-c (mmol/L)	1.36 ± 0.36	1.69 ± 0.99	1.53 ± 0.57
Urea (mmol/L)	7.62 ± 3.17^ab^	4.62 ± 1.11	4.44 ± 1.02
Creatinine (μmol/L)	88.39 ± 43.51	60.90 ± 14.79	70.76 ± 16.66
Glucose (mmol/L)	6.35 ± 1.53	5.61 ± 1.47	5.58 ± 1.27
Uric acid (μmol/L)	406.90 ± 77.61^ab^	328.5 ± 62.33	321.90 ± 85.13
AST (U/L)	18.80 ± 3.55	19.90 ± 7.61	19.50 ± 4.45
ALT (U/L)	18.07 ± 4.08	20.30 ± 6.96	20.60 ± 7.44
ESR (mm/h)	26.00 ± 17.8^a^	20.50 ± 11.61	13.40 ± 3.81
CRP (mg/L)	5.28 ± 4.38^a^	2.55 ± 2.28	1.84 ± 0.89

a *p* < 0.05 vs. HCs.

b *p* < 0.05 vs. BA patients.

### Plasma metabolomic profiling discriminates EGPA from BA and HC

**Fig 1:**
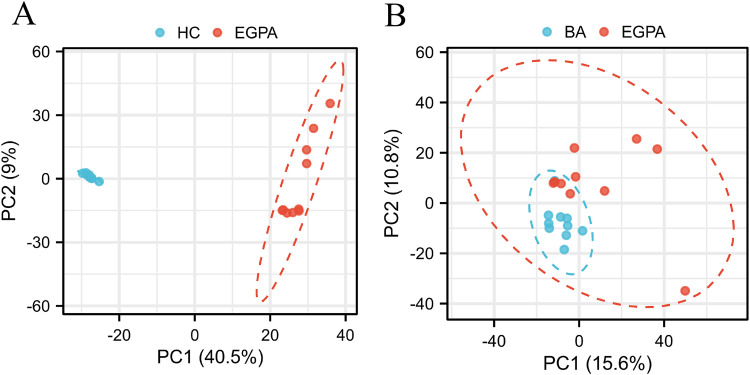
PCA score plot. (A) EGPA patients (red) versus HCs (blue). (B) EGPA patients (red) versus BA patients (blue).

**Fig 2:**
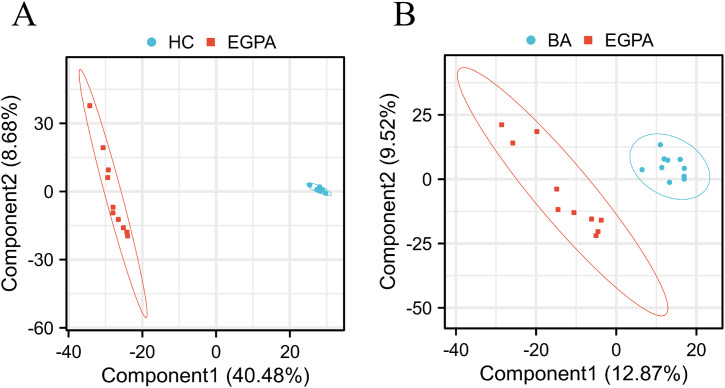
OPLS-DA score plot. (A) EGPA patients (red) versus HCs (blue). (B) EGPA patients (red) versus BA patients (blue).

Metabolomic profiling revealed distinct separations among the three groups. The PCA score plot (Fig 1) revealed distinct clustering of metabolites between EGPA patients, BA patients, and HCs. The resulting score plot demonstrated a significant separation between EGPA patients and HCs (Fig 2A). The predictive component demonstrated excellent explanatory and predictive power(R²X = 0.483, R²Y = 0.991, Q² = 0.987), while the orthogonal component captured only 8.8% of the variation(R²X = 0.088, R²Y = 0.007, Q² = 0.006), confirming that the separation was robust and not driven by confounding factors.

For the comparison between EGPA and BA patients, the OPLS-DA model also revealed a separation (Fig 2B). The predictive component yielded R²X = 0.093, R²Y = 0.810, and Q² = 0.475, indicating moderate explanatory and predictive ability. The orthogonal component showed R²X = 0.127, R²Y = 0.153, and Q² = 0.046. The Q² value of 0.475 suggested acceptable predictive performance.

### Pathway profiling in EGPA patients versus HCs

**Fig 3:**
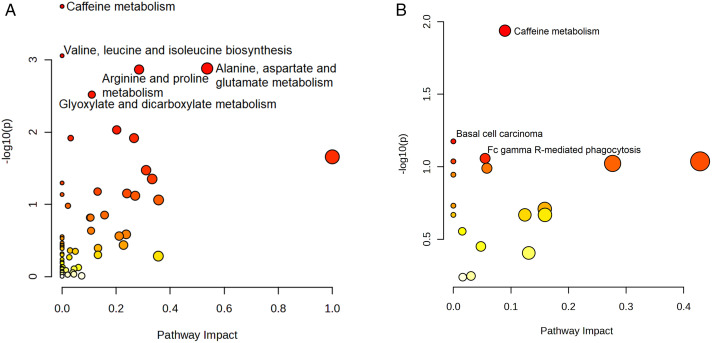
Bubble plot of KEGG pathway enrichment. (A) EGPA patients versus HCs. (B) EGPA patients versus BA patients.

A total of 971 metabolites were significantly differentially expressed between EGPA patients and HCs. Pathway enrichment analysis showed that 59 enriched pathways were identified from the KEGG database, five of which were statistically significant (P < 0.05). As illustrated in the bubble plot (Fig 3A), these pathways were caffeine metabolism; valine, leucine and isoleucine biosynthesis; alanine, aspartate and glutamate metabolism; arginine and proline metabolism; and glyoxylate and dicarboxylate metabolism. Caffeine metabolism showed the highest significance (P = 0.0002, 5 mapped metabolites). Detailed enrichment results for five pathways, including impact scores, P-values, and metabolite counts, were provided in S1 Table.

Further analysis of these five significant pathways revealed 24 metabolites that contributed to the enrichment, including amino acids, organic acids, and caffeine derivatives. The complete list of these 24 metabolites, along with their FC and P- values, was provided in S3 Table.

### Pathway profiling in EGPA patients versus BA patients

A total of 161 metabolites were identified as significantly altered between EGPA and BA patients. Pathway enrichment analysis showed that 102 enriched pathways were identified from KEGG database, three of which were statistically significant (P < 0.05). As shown in Fig 3B, these pathways were caffeine metabolism; basal cell carcinoma; and Fc gamma R-mediated phagocytosis. Among them, caffeine metabolism was the most significantly altered pathway (P = 0.002). Detailed enrichment results for the three pathways, including impact scores, P-values, and metabolite counts, were provided in S2 Table.

Further analysis of these three significant pathways identified five key metabolites contributing to the enrichment, namely 5-acetylamino-6-amino-3-methyluracil, 5-acetylamino-6-formylamino-3-methyluracil, 3-methylxanthine, cholesterol, and arachidonic acid. Detailed information was provided in S4 Table.

**Fig 4:**
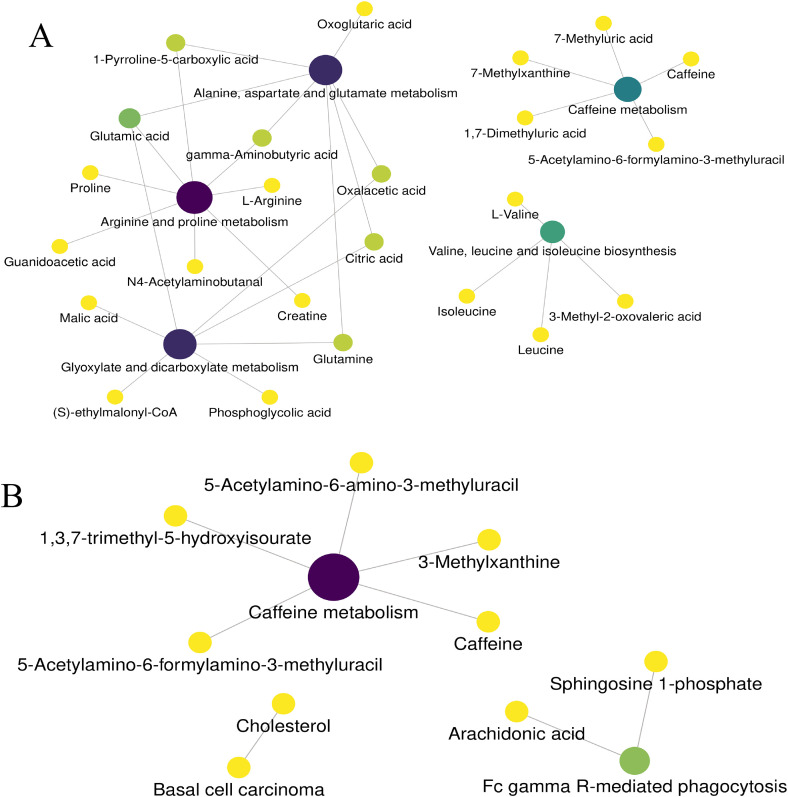
Network of the enriched pathways. (A) EGPA patients versus HCs. (B) EGPA patients versus BA patients.

A comprehensive network of all enriched pathways was presented in Fig 4, where nodes represent metabolite sets, with color indicating the P value and node size corresponding to the fold enrichment.

### Metabolomic biomarkers for diagnosing EGPA

We evaluated the diagnostic accuracy of the identified biomarkers using ROC curve analysis. Among the 24 metabolites derived from the five significant pathways mentioned above, 21 demonstrated effective discriminatory power (AUC > 0.8) in distinguishing EGPA patients from HCs. As shown in Table 3, γ-aminobutyric acid and creatinine had AUC values below 0.9, whereas the remaining biomarkers exhibited AUC values exceeding 0.9, indicating high diagnostic potential for EGPA.

**Table 3:** ROC analysis of candidate biomarkers for distinguishing EGPA.

Biomarker	AUC	95% CI
L-Proline	1.00	1.000–1.000
γ-Aminobutyric acid	0.83	0.640–1.000
N4-Acetylaminobutanal	1.00	1.000–1.000
7-Methylxanthine	1.00	1.000–1.000
Creatine	0.85	0.666–1.000
1-Pyrroline-5-carboxylic acid	1.00	1.000–1.000
L-Glutamic acid	0.93	0.820–1.000
1,7-Dimethylurate	1.00	1.000–1.000
L-Valine	1.00	1.000–1.000
L-Isoleucine	0.95	0.860–1.000
L-Leucine	0.96	0.875–1.000
Caffeine	1.00	1.000–1.000
(S)-Ethylmalonyl-CoA	0.93	0.790–1.000
FA 4:2;O3	1.00	1.000–1.000
L-Glutamine	1.00	1.000–1.000
Citric acid	1.00	1.000–1.000
DL-Malic acid	1.00	1.000–1.000
5-Acetylamino-6-formylamino-3-methyluracil	1.00	1.000–1.000
L-Arginine	1.00	1.000–1.000
Oxoglutaric acid	1.00	1.000–1.000
3-Methyl-2-oxovalerate	0.96	0.875–1.000

### Metabolomic biomarkers for differential diagnosis of EGPA and BA

Four of the five metabolites derived from the key pathways mentioned above demonstrated high diagnostic potential (AUC > 0.8) and were therefore identified as candidate plasma biomarkers for the differential diagnosis of EGPA and BA, as described in Table 4. These four biomarkers were cholesterol, 5-acetylamino-6-formylamino-3-methyluracil, 5-acetylamino-6-amino-3-methyluracil, and 3-methylxanthine.

**Table 4:** ROC analysis of candidate biomarkers for differentiating EGPA from BA.

Biomarker	AUC	95% CI
Cholesterol	0.96	0.886–1.000
5-Acetylamino-6-formylamino-3-methyluracil	0.86	0.696–1.000
5-Acetylamino-6-amino-3-methyluracil	0.86	0.686–1.000
3-Methylxanthine	0.89	0.736–1.000

## Discuss

Metabolomics analysis based on LC/MS platforms had been increasingly recognized as a valuable tool for disease diagnosis and biomarker discovery [17–19]. To our knowledge, this study was the first application of UPLC-QTOF/MS-based untargeted metabolomics to characterize plasma metabolic profiles in patients with EGPA in comparison to both BA patients and HCs.

Our results demonstrated that the LC/MS-based metabolomics approach effectively differentiated EGPA patients from BA patients and HCs according to their differential plasma metabolite profiles. This discrimination was demonstrated by the clear separation of the plasma spectral data through PCA and OPLS-DA modeling (Fig 1, Table 3). We identified 24 differentially expressed metabolites (FC > 2.0 or < 0.5, nominal P < 0.05) between EGPA patients and HCs. These metabolites were primarily enriched in five key pathways: caffeine metabolism; valine, leucine and isoleucine biosynthesis; alanine, aspartate and glutamate metabolism; arginine and proline metabolism; and glyoxylate and dicarboxylate metabolism. These findings identified candidate metabolomic signatures associated with EGPA.

EGPA often involved the peripheral nervous system and induced complications such as polyneuritis. Recent studies reported that γ-aminobutyric acid (GABA) suppressed inflammation, in part by reducing the production of proinflammatory mediators [20]. The imbalance of GABA homeostasis could disrupt the function of the neuro-immune axis. We observed a significant elevation of L-glutamic acid in patients with EGPA. This finding was consistent with previous reports in multiple sclerosis (MS) [21], suggesting a potential link to glutamatergic dysfunction that may be relevant to both diseases. In the present study, GABA was downregulated and L-glutamic acid was upregulated in EGPA patients. These findings raised the hypothesis that the imbalance between these two metabolites may be associated with the eosinophilic inflammation and vascular damage characteristic of EGPA, although the exact mechanisms require further investigation.

In our study, we observed significantly elevated levels of branched-chain amino acids (BCAAs), including L-valine, L-isoleucine, and L-leucine, in EGPA patients compared to HCs. They were known to be involved not only in protein synthesis, but also in regulating immune cell proliferation and metabolic reprogramming. Dysregulation of BCAA metabolism was reported in autoimmune diseases, including rheumatoid arthritis and systemic vasculitis [22], suggesting a potential link between BCAA homeostasis and inflammatory conditions.

3-Methyl-2-oxovalerate, a critical intermediate in the isoleucine catabolic pathway, was also elevated in EGPA patients. Studies found that 3-methyl-2-oxovalerate was the key differential metabolite in rheumatoid arthritis (RA) patients, with serum levels showing a continuous increase during anti-TNF therapy [23]. The presence of elevations in both BCAAs and their catabolic intermediate raised the possibility of disrupted BCAA metabolism in EGPA.

Glutamine and proline are interconverted through Δ^1^-pyrroline-5-carboxylate (P5C). Glutamine metabolism was known to serve as an energy source for immune cell activation, and eosinophils similarly relied on amino acid metabolism to sustain their proliferation and effector functions during inflammation [24]. Previous studies reported that P5C could suppress T cell signaling by increasing reactive oxygen species (ROS) and SHP1, while decreasing cytokines and ATP production [25]. Additionally, glutamine metabolism was reported to critically regulate eosinophil activation, especially its important regulatory significance in the production of eosinophil-derived inflammatory cytokines [26]. Cellular experiments showed that proline treatment downregulated T helper 2 (Th2)-related cytokines (IL-4 and IL-13), while upregulating regulatory T-cell related cytokines (IL-10 and transforming growth factor-β) [27]. The increased levels of glutamine, proline, and Δ1-pyrroline-5-carboxylate (P5C) observed in our study supported the hypothesis that disruptions in this metabolic pathway may be associated with immune dysregulation in EGPA. Further functional studies are required to confirm this hypothesis.

Research suggested that GABA was the downstream metabolite of N4-acetylaminobutanal and inhibited the autoimmune activity of T cells by binding to GABA-A receptors on T cell surfaces [28]. This finding was consistent with the previously reported immunomodulatory effects of GABA. As far as we know, this study was the first to identify an association between N4-acetylaminobutanal and EGPA.

Caffeine was mainly metabolized by cytochrome P450 1A2 (CYP1A2) to produce metabolites such as 7-methylxanthine, 1,7-dimethyluric acid, and 5-acetylamino-6-formylamino-3-methyluracil [29]. In the present study, we observed significantly lower levels of caffeine and its metabolites in EGPA patients compared to HCs. This observation was supported by previous studies that caffeine exerted anti-inflammatory effects by inhibiting the phagocytic function of mononuclear phagocytes and reducing the production of TNF-α and other proinflammatory cytokines through the cAMP/PKA pathway [30]. Previous studies demonstrated that systemic inflammation and inflammatory mediators such as interleukin-6 can modulate the catalytic activity of CYP1A2 [31]. Considering that EGPA was a systemic autoimmune vasculitis characterized by prominent systemic inflammation, whereas BA mainly represented a localized allergic airway inflammation, we speculated that the distinct inflammatory microenvironments may partially contribute to differences in CYP1A2 activity and thus the caffeine metabolic profile. However, we acknowledge that potential confounding factors, including dietary caffeine intake, lifestyle, smoking status, and other environmental exposures, cannot be fully excluded. Further studies with standardized documentation of dietary and lifestyle habits are warranted to verify the contribution of CYP1A2 activity to the observed metabolomic differences between EGPA and BA.

In pro-inflammatory macrophages, citrate was transported to the cytoplasm via carrier SLC25A1, and ATP-citrate lyase (ACLY) catalyzed the production of acetyl-CoA. This metabolic pathway was reported to promote the synthesis of proinflammatory mediators, including nitric oxide (NO), reactive oxygen species (ROS), and prostaglandin E_2_ (PGE_₂_), potentially contributing to vascular endothelial injury [32]. Previous studies indicated that abnormal citrate metabolism was not only positively correlated with disease activity of systemic vasculitis, but that pro-inflammatory metabolic reprogramming mediated by citrate metabolism might also exacerbate immune-mediated vascular injury. In the present study, citrate levels were significantly higher in EGPA patients than in HCs. Notably, several metabolites altered in our EGPA cohort overlapped with those previously reported in other vasculitis or inflammatory conditions [33]. This overlap suggested potential common metabolic perturbations associated with vascular injury across different diseases.

In our study, we observed elevated levels of (S)-ethylmalonyl-CoA and FA 4_2;O3 in EGPA patients compared to healthy controls, suggesting perturbations in lipid metabolic pathways. Lipid rafts of synovial macrophages in rheumatoid arthritis (RA) could recruit pattern recognition receptors such as TLR4, enhanced LPS-mediated activation of NF-κB, and exacerbated joint inflammation. The levels of cholesterol and glycosphingolipid (GSL) in lipid rafts of T cells and B cells in patients with systemic lupus erythematosus (SLE) were increased, which led to the excessive activation of TCR/BCR signaling and promoted the proliferation of autoreactive lymphocytes and the production of autoantibodies [34].

Our findings revealed significantly lower plasma cholesterol levels in EGPA patients compared to BA patients. Recent studies reported that adult asthma patients had significantly increased levels of TC, LDL-C, and TG compared with healthy people. Among them, TC and LDL-C levels were positively correlated with the frequency of asthma acute attack, suggesting a link between lipid metabolism and the activity of asthma [35]. In autoimmune vasculitis, it was suggested that the transport rate of cholesterol to the vascular wall increased by 30%−50% due to vascular endothelial injury. At the same time, it was proposed that the cholesterol uptake rate of activated vasculitis-related immune cells (such as eosinophils and macrophages) was significantly higher than that of asthma-related immune cells (such as Th2 cells), which could contribute to the reduction of peripheral blood cholesterol levels [34].

In the present work, we observed that the lower levels of caffeine metabolites, including 5-acetylamino-6-formylamino-3-methyluracil (AFMU), 5-acetylamino-6-amino-3-methyluracil (AAMU), and 3-methylxanthine, produced by CYP1A2, were significantly different between patients with EGPA and BA [36]. This observation raised the possibility that CYP1A2 enzyme activity may differ between the two groups, contributing to a differential caffeine metabolic phenotype. One potential explanation for this difference related to medication effects. Conventional asthma drugs had no significant inhibition on the activities of caffeine metabolism enzymes such as CYP1A2. However, exogenous interferons, which may be used in the treatment of EGPA, could inhibit the gene expression and enzyme activity of hepatic CYP1A2 through the JAK-STAT pathway [37]. This difference might partly explain the lower levels of caffeine metabolites seen in EGPA patients. Collectively, these metabolites may serve as candidate biomarkers to differentiate EGPA from BA, particularly in cases with overlapping eosinophilic airway symptoms. Future studies are needed to validate these findings in larger cohorts, to clarify the potential role of medication effects on the observed metabolic differences.

To our knowledge, this study was the first to systematically characterize metabolite alterations across multiple pathways in patients with EGPA. Our findings identified dysregulations of the glutamate-glutamine metabolic axis, purine metabolism, and BCAA metabolism as prominent features associated with EGPA. Beyond providing novel insights into the immune-metabolic regulatory network associated with EGPA, these differential metabolites represented candidate biomarkers for this disease. Importantly, we observed differential levels of cholesterol, 5-acetylamino-6-formylamino-3-methyluracil, 5-acetylamino-6-amino-3-methyluracil, and 3-methylxanthine between EGPA and BA patients, suggesting distinct metabolic profiles associated with these two conditions. These findings raised the possibility that cholesterol-related metabolites might reflect differences between systemic and localized inflammatory processes, whereas purine metabolites and 3-methylxanthine might discriminate between autoimmune and allergic inflammatory pathways. Collectively, these metabolites might serve as candidate biomarkers to differentiate EGPA from BA, particularly in cases with overlapping eosinophilic airway manifestations.

Our study had several inherent limitations that should be acknowledged. First, the correlation between alterations in metabolite levels and EGPA disease activity, as well as ANCA positivity status, remained to be clarified. Second, cellular and animal experimental evidence was lacking to validate the functional roles of these differential metabolites on immune-inflammatory responses. Third, the small sample size might have reduced the statistical power of the current findings and increased the risk of false positives,

To address these limitations and extend the present findings, future studies should prioritize expanding the sample size and performing stratified analyses according to detailed clinical phenotypes (e.g., disease activity, ANCA status, and organ involvement). Additionally, in vitro experiments are warranted to investigate the functional roles of key metabolites in eosinophils, T cells, and other immune cells implicated in EGPA pathogenesis. Furthermore, exploring the potential therapeutic value of targeted metabolic pathway inhibitors—such as glutaminase inhibitors and xanthine oxidase inhibitors—may provide novel strategies for EGPA management.

## Supporting information

S1 Table Significantly enriched metabolic pathways in EGPA vs. HC. (DOCX)

S2 Table Significantly enriched metabolic pathways in EGPA vs. BA. (DOCX)

S3 Table Differentially Expressed Metabolites in EGPA vs. HC. (DOCX)

S4 Table Differentially Expressed Metabolites in EGPA vs. BA. (DOCX)

S1 File Supporting information. (XLSX)
