# Supplementary material for: Identification of a diagnostic metabolomic fingerprint in plasma for eosinophilic granulomatosis with polyangiitis
Source: PLoS One. 2026 May 12;21(5):e0343182. doi: 10.1371/journal.pone.0343182 (PMC13166926; doi:10.1371/journal.pone.0343182)
Supplement: S1 Table — (DOCX) [file pone.0343182.s001.docx]

| Table S1 Significantly enriched metabolic pathways in EGPA vs. HCs | | | | |
| --- | --- | --- | --- | --- |
| **Pathway Name** | **Total Metabolites** | **Hits (Matched)** | **P-value** | **Pathway Impact** |
| Caffeine metabolism | 10 | 5 | 0.000 | 0.000 |
| Valine, leucine and isoleucine biosynthesis | 8 | 4 | 0.001 | 0.000 |
| Alanine, aspartate and glutamate metabolism | 28 | 7 | 0.001 | 0.537 |
| Arginine and proline metabolism | 36 | 8 | 0.001 | 0.285 |
| Glyoxylate and dicarboxylate metabolism | 32 | 7 | 0.003 | 0.110 |
