# Supplementary material for: Identification of a diagnostic metabolomic fingerprint in plasma for eosinophilic granulomatosis with polyangiitis
Source: PLoS One. 2026 May 12;21(5):e0343182. doi: 10.1371/journal.pone.0343182 (PMC13166926; doi:10.1371/journal.pone.0343182)
Supplement: S2 Table — (DOCX) [file pone.0343182.s002.docx]

| Table S2 Significantly enriched metabolic pathways in EGPA vs. BA | | | | |
| --- | --- | --- | --- | --- |
| **Pathway Name** | **Total Metabolites** | **Hits (Matched)** | **P-value** | **Pathway Impact** |
| Caffeine metabolism | 22 | 5 | 0.002 | 0.090 |
| Basal cell carcinoma | 1 | 1 | 0.045 | 0.000 |
| Fc gamma R-mediated phagocytosis | 8 | 2 | 0.047 | 0.055 |
