# Supplementary material for: Identification of a diagnostic metabolomic fingerprint in plasma for eosinophilic granulomatosis with polyangiitis
Source: PLoS One. 2026 May 12;21(5):e0343182. doi: 10.1371/journal.pone.0343182 (PMC13166926; doi:10.1371/journal.pone.0343182)
Supplement: S3 Table — (DOCX) [file pone.0343182.s003.docx]

Supplementary Table S3 Differentially Expressed Metabolites in EGPA vs. HC

| **No** | **m/z** | **tR**  **(min)** | **Formula** | **Identiﬁed metabolites** | **FC** | **p-value** | **VIP** | **KEGG** |
| --- | --- | --- | --- | --- | --- | --- | --- | --- |
| 1 | 255.07 | 287.8 | C7H8N4O3 | 1,7-dimethylurate | 17.30 | 2.20E-10 | 9.66 | C16356 |
| 2 | 132.10 | 98.9 | C6H13NO2 | L-Isoleucine | 4.86 | 1.59E-02 | 1.80 | C00407 |
| 3 | 132.10 | 135.5 | C6H13NO2 | L-Leucine | 9.67 | 2.67E-03 | 2.57 | C00123 |
| 4 | 118.09 | 118.2 | C5H11NO2 | L-Valine | 1297.20 | 4.37E-04 | 3.36 | C00183 |
| 5 | 265.02 | 40.1 | C4H4O5 | FA 4_2;O3 | 5.04 | 1.56E-05 | 4.81 | C00036 |
| 6 | 114.05 | 68.4 | C5H7NO2 | 1-Pyrroline-5-carboxylic acid | 3.94 | 1.52E-05 | 4.82 | C03912 |
| 7 | 148.06 | 50.1 | C5H9NO4 | L-Glutamic acid | 3.54 | 2.42E-03 | 2.62 | C00025 |
| 8 | 118.06 | 49.4 | C3H7N3O2 | Guanidinoacetate | 6.49 | 3.58E-02 | 1.45 | C00581 |
| 9 | 132.08 | 49.5 | C4H9N3O2 | Creatine | 2.25 | 2.90E-02 | 1.54 | C00300 |
| 10 | 130.09 | 52.4 | C6H11NO2 | N4-Acetylaminobutanal | 5.08 | 5.90E-04 | 3.23 | C05936 |
| 11 | 116.07 | 50.2 | C5H9NO2 | L-Proline | 734.99 | 1.17E-07 | 6.93 | C00148 |
| 12 | 156.99 | 344.6 | C2H5O6P | Phosphoglycolic acid | 2.41 | 1.78E-02 | 1.75 | C00988 |
| 13 | 104.07 | 50.2 | C4H9NO2 | γ-Aminobutyric acid | 0.34 | 9.87E-03 | 2.01 | C00334 |
| 14 | 195.09 | 265.8 | C8H10N4O2 | Caffeine | 0.13 | 7.70E-09 | 8.11 | C07481 |
| 15 | 882.16 | 279.6 | C26H42N7O19P3S | (S)-ethylmalonyl-CoA | 4.09 | 1.24E-02 | 1.91 | C18026 |
| 16 | 225.06 | 49.5 | C8H10N4O4 | 5-Acetylamino-6-formylamino-3-methyluracil | 28.53 | 1.36E-04 | 3.87 | C16365 |
| 17 | 165.04 | 408.9 | C6H6N4O2 | 7-Methylxanthine | 22.72 | 4.38E-12 | 11.36 | C16353 |
| 18 | 129.05 | 100.6 | C6H10O3 | 3-methyl-2-oxovalerate | 5.74 | 1.26E-03 | 2.90 | C00671 |
| 19 | 191.02 | 38.4 | C6H8O7 | Citric acid | 1715.50 | 8.96E-09 | 8.05 | C00158 |
| 20 | 145.01 | 42 | C5H6O5 | Oxoglutaric acid | 5.77 | 8.14E-07 | 6.09 | C00026 |
| 21 | 145.06 | 46.8 | C5H10N2O3 | L-Glutamine | 5.39 | 3.40E-07 | 6.47 | C00064 |
| 22 | 173.10 | 66.1 | C6H14N4O2 | L-Arginine | 16.23 | 1.43E-03 | 2.84 | C00062 |
| 23 | 133.01 | 41.2 | C4H6O5 | DL-Malic acid | 31.49 | 1.09E-04 | 3.96 | C00149 |
| 24 | 181.04 | 46.5 | C6H6N4O3 | 7-Methyluric acid | 0.45 | 1.05E-02 | 1.98 | C16355 |
