# Supplementary material for: Identification of a diagnostic metabolomic fingerprint in plasma for eosinophilic granulomatosis with polyangiitis
Source: PLoS One. 2026 May 12;21(5):e0343182. doi: 10.1371/journal.pone.0343182 (PMC13166926; doi:10.1371/journal.pone.0343182)
Supplement: S4 Table — (DOCX) [file pone.0343182.s004.docx]

Supplementary Table S4 Differentially Expressed Metabolites in EGPA vs. BA

| **No** | **m/z** | **tR**  **(min)** | **Formula** | **Identiﬁed metabolites** | **FC** | **p-value** | **VIP** | **KEGG** |
| --- | --- | --- | --- | --- | --- | --- | --- | --- |
| 1 | 179.06 | 66.6 | C7H10N4O3 | 5-Acetylamino-6-amino-3-methyluracil | 0.45 | 2.59E-02 | 1.70 | C16366 |
| 2 | 225.06 | 49.5 | C8H10N4O4 | 5-Acetylamino-6-formylamino-3-methyluracil | 0.49 | 4.79E-03 | 2.04 | C16365 |
| 3 | 225.06 | 66.7 | C6H6N4O2 | 3-Methylxanthine | 0.44 | 1.39E-02 | 1.90 | C16357 |
| 4 | 369.35 | 411 | C27H46O | Cholesterol | 0.16 | 2.48E-04 | 2.31 | C00187 |
| 5 | 303.23 | 450 | C20H32O2 | Arachidonic acid | 0.39 | 8.29E-03 | 1.91 | C00219 |
